# Abdominal Pain in Children

**Published:** 1921-04-02

**Authors:** 


### MINOR MEDICAL PROBLEMS.

### Abdominal Pain in Children.

It is a commonly appreciated fact that abdominal pain in a child may arise from nothing more serious than a green apple and an attack of painful indigestion, whereas the same symptom may indicate the onset of a peritonitis arising from an acutely inflamed appendix. And it is not always easy for a practitioner diagnostically to steer a safe middle course when there lies, on the one hand, a natural anxiety not to overlook the seriousness of a case and, on the other, an excusable reluctance unduly to alarm the parents.

### "Extra-Abdominal" Causes.

As Robert Hutchison emphatically points out in a recent contribution to this subject, the difficulty is increased by the circumstance that, though the position of an early, ill-defined pain may point to abdominal disease, the seat of the trouble may lie in an entirely different system. It is therefore well to bear in mind the "extra-abdominal" causes, and the author already quoted gives us a useful classification according to two chief localities—the abdominal wall and the thorax. Among those causes in the abdominal wall may be placed spinal caries, lateral curvature, rheumatism, ruptures, hip disease, and herpes zoster. All these conditions give rise to classical signs if only they are carefully looked for, and abdominal pain, particularly recurrent pain, should put the practitioner on his guard. Perhaps the commonest diagnostic error occurs when a case of pneumonia at the base of the right lung is mistaken for one of appendicitis. Early pneumonia in childhood is frequently accompanied by vomiting, and this helps still further to simulate the picture of abdominal disease. It is suggested that the abdominal pain is present only when the diaphragmatic pleura is involved, and in this connection it is interesting to note that in pericarditis when the diaphragmatic surface is definitely affected, a typical attack of abdominal pain is produced.

Apparently there are sundry less common thoracic conditions which may act in the same way, yet in childhood pneumonia and pericarditis are the doctor's chief consideration, and will here serve as being sufficiently illustrative.

### The "Acute Abdomen."

Coming next to the truly intra-abdominal causes of pain, Hutchison divides them for convenience into those producing sudden or "catastrophic" pain and those giving the symptom recurrently or chronically. By the former the author implies those instances in which pain sets in both unexpectedly and with great intensity. Fortunately, however, the possible reasons for the "acute abdomen" are far fewer in the child than in the adult.

Confronted with sudden severe abdominal pain in a child one has, apart from the green apple, to consider appendicitis or some form of acute intestinal obstruction, and by far the commonest is the typical intussusception, where one portion of the intestine slips into an adjacent portion forming a firmly fixed invagination. Irregular peristalsis, the direct cause of the condition, is obviously common in young, carelessly-fed children. The elongated tumour produced by this process and by the consequent constriction of the mesenteric vessels can, in most cases, be palpated if it be searched for carefully, and the engorgement of the gut, with resultant blood in the evacuations, is of first-rate diagnostic value.

On the other hand, chronic and recurrent abdominal pains in the child are open to more varied explanations. Rarely are they traceable to the stomach, the intestines being usually the seat of primary trouble. Here Hutchison divides the possibilities into (*a*) ordinary uncomplicated colic; (*b*) "umbilical" colic; (*c*) enterospasm; and (*d*) chronic obstruction; but this differentiation is not so important as the simple dietetic and hygienic revision in the patient's daily life which the practitioner is called upon to institute. It is necessary, in this connection, to realise that, apart from an ill-balanced diet, the child may be relatively deficient in its power to digest one or other of the chief food factors, such as carbohydrates, and much valuable information may be obtained from inspection of the stools by a means more certain than a casual inspection. The low-power microscope will reveal the cause of infantile colic with surprising frequency.

### Exceptional Cases.

Nevertheless, it is unjustifiable to expect every case to have so simple an origin. The closest clinical observation is required in elimination of the appendix as a local source of trouble, and no penny-in-the-slot method at present exists to help us. Also one must bear in mind that enlarged mesenteric glands more commonly give rise to abdominal pain than is generally realised. The various affections, particularly bacterial infections, of the urinary tract, are a frequent and often overlooked cause of abdominal pain in early life. Movable kidney and attacks of true renal colic are rarely to be considered except in later years, but it must be remembered that kinking of the ureter and small ureteric calculi are of relatively frequent occurrence during childhood. Overdistension of the bladder is another possible cause. In the male this may be actively produced by phimosis or a narrow meatus, and attacks of hypogastric pain from this cause are relatively common. In the opposite sex, salpingitis is not to be forgotten, even though the patient be of tender years. And so might one proceed to many other minor causes and possibilities, but enough will have been said in this note if in its outline there lies the lesson that simple and unimportant though a case of infantile colic may appear to be, yet there are few conditions which call for greater care and more acute observation on the part of the family practitioner.

